# A Sleep Questionnaire for Children with Severe Psychomotor Impairment (SNAKE)—Concordance with a Global Rating of Sleep Quality

**DOI:** 10.3390/children5020020

**Published:** 2018-02-01

**Authors:** Larissa Alice Dreier, Boris Zernikow, Markus Blankenburg, Julia Wager

**Affiliations:** 1 Paediatric Palliative Care Centre, Children’s and Adolescents’ Hospital, 45711 Datteln, Germany; l.dreier@kinderpalliativzentrum.de (L.A.D.); b.zernikow@deutsches-kinderschmerzzentrum.de (B.Z.); 2 Department of Children’s Pain Therapy and Paediatric Palliative Care, Faculty of Health, School of Medicine, Witten/Herdecke University, 58448 Witten, Germany; m.blankenburg@klinikum-stuttgart.de; 3 Paediatric Neurology, Psychosomatics and Pain Therapy, Center for Child, Youth and Women’s Health, Klinikum Stuttgart, Olgahospital/Frauenklinik, 70174 Stuttgart, Germany

**Keywords:** sleep, pediatric, SNAKE, life-limiting, neurological, impairments

## Abstract

Sleep problems are a common and serious issue in children with life-limiting conditions (LLCs) and severe psychomotor impairment (SPMI). The “Sleep Questionnaire for Children with Severe Psychomotor Impairment” (Schlaffragebogen für Kinder mit Neurologischen und Anderen Komplexen Erkrankungen, SNAKE) was developed for this unique patient group. In a proxy rating, the SNAKE assesses five different dimensions of sleep(-associated) problems (disturbances going to sleep, disturbances remaining asleep, arousal and breathing disorders, daytime sleepiness, and daytime behavior disorders). It has been tested with respect to construct validity and some aspects of criterion validity. The present study examined whether the five SNAKE scales are consistent with parents’ or other caregivers’ global ratings of a child’s sleep quality. Data from a comprehensive dataset of children and adolescents with LLCs and SPMI were analyzed through correlation coefficients and Mann–Whitney U testing. The results confirmed the consistency of both sources of information. The highest levels of agreements with the global rating were achieved for disturbances in terms of going to sleep and disturbances with respect to remaining asleep. The results demonstrate that the scales and therefore the SNAKE itself is well-suited for gathering information on different sleep(-associated) problems in this vulnerable population.

## 1. Introduction

Referring to data from the UK, approximately 32 per 10,000 children and adolescents suffer from life-limiting conditions (LLCs), with a rising trend [1]. Accordingly, genetic, neurological, or metabolic diseases that are accompanied by severe psychomotor impairments (SPMI) constitute the most represented diagnoses [2,3].

With an assumed prevalence of 60–80%, sleep problems are a common issue in these children and adolescents [4]. In accordance with the “International Classification of Sleep disorders” (ICSD-2; [5]), sleep problems can generally be categorized into excessive sleepiness, sleeplessness, and behavioral disturbances [6]. There are several studies that confirm the relationship between severe chronic illnesses and sleep disturbances, such as difficulties in initiating and maintaining sleep [7,8,9,10], sleep-associated respiratory problems [4,11,12], daytime sleepiness [13], parasomnia [14,15], and irregular sleep–wake rhythm [16,17]. Pediatric sleep problems burden both the children themselves and their caregivers [18,19]. As a result, parents are commonly affected by psychological and somatic problems that, in turn, negatively influence their child’s care [18]. However, the etiology of sleep problems in children with LLCs and SPMI is not entirely clear [20,21]. To address this issue and to support an efficient diagnosis of sleep problems, reliable and valid measures are essential [4].

There are a number of sleep measures specifically developed for pediatric use. For instance, the “Sleep Disturbance Scale for Children” (SDSC; [22,23]), the “Children’s Sleep Habits Questionnaire” (CSHQ; [14,24,25,26,27]) and the “Sleep Behavior Questionnaire” (SBQ; [28,29]) have been applied in numerous investigations [11]. However, these questionnaires were originally developed for use in healthy children and thus do not meet the special requirements of children with SPMI who commonly suffer from impaired language capability [21]. To address this problem, the “Sleep Questionnaire for Children with Severe Psychomotor Impairment” (Schlaffragebogen für Kinder mit Neurologischen und Anderen Komplexen Erkrankungen, SNAKE) was developed [21]. The SNAKE is a multidimensional and comprehensive proxy assessment that takes medical as well as psychosocial aspects of sleep problems in children with LLCs and SPMI into account. Its five scales are based on the ICSD-2 [5] and cover different sleep(-related) problems. Further, several items ask directly about sleep issues that may emerge from conditions linked to LLCs (for example medical care, need for repositioning, pain, epileptic seizures, breathing difficulties) and therefore are unique for the vulnerable population of severely disabled children and adolescents. Even though the SNAKE’s reliability, its construct validity, and some aspects of criterion validity have been confirmed [21], the relationship between the scale’s ratings and the caretakers’ global assessment of the child’s sleep quality has not yet been examined. The present paper addresses the question of how consistent the scores obtained in the SNAKE scales are with the caretaker’s global rating. Concordance between the SNAKE scales and the global rating would be an additional confirmation of the questionnaire’s validity and its practical benefit as a whole.

## 2. Materials and Methods

### 2.1. Dataset

Data were derived from a comprehensive dataset of *N* = 226 children and adolescents aged 1–25 years (*M* = 10.39) who had been diagnosed with LLCs and SPMI. Participants and their parents were originally recruited from one outpatient facility and three inpatient institutions within the scope of another study [21]. Ethical approval was obtained through the Ethics Committee of the Children’s and Adolescent’s Hospital Datteln, Germany (Approval code 2008/06/26/MB). Informed consent was obtained from all participants. Research ethics of this investigation comply fully with the Declaration of Helsinki and the German data protection law. A number of studies based on the same dataset have been published [18,21,30].

### 2.2. The Sleep Questionnaire for Children with Severe Psychomotor Impairment (SNAKE)

One challenge for the SNAKE is that the use of self-rating questionnaires in children with LLCs and SPMI is hindered by impaired cognitive and communicative abilities [21]. Hence, the SNAKE is based on proxy reporting by parents or other caregivers on behalf of their child with respect to the previous four weeks [21]. It consists of 54 items. Of these, 23 items belong to one of the following five scales:

- Disturbances going to sleep
- Disturbances remaining asleep
- Arousal and breathing disorders
- Daytime sleepiness
- Daytime behavior disorders

The remaining 31 items gather information on, for instance, sleep conditions, sleep duration, and efficacy, the core characteristics of the child and his or her family, and aspects of general sleep quality that do not feed into one of the SNAKE scales [21]. Core characteristics are the child’s age, sex, weight, height, diagnosis or diagnoses, medication(s), the parents’ marital status, where the child predominantly lives, the number of children in the household, the number of people in the household, and who filled out the questionnaire.

A confirmatory factor analysis conducted as part of the initial validation study showed a good fit. Test/retest reliability of the five factors (*r^rt^* > 0.7) and internal consistencies (Cronbach’s alpha > 0.7) are high [21]. The total score of a scale can be derived from the addition of its raw scores. For interpretation, a higher score on a particular scale corresponds with more problematic sleep or sleep-related behavior [31]. The SNAKE is available in English and German and can be requested free of charge as a download from the German Pediatric Pain Center and Pediatric Palliative Care Center [32].

### 2.3. Global Rating of Sleep Quality

The exact wording of the item asking for a global rating of the child’s sleep is: “How would you rate your child’s sleep quality overall?” The question can be rated by parents or other caregivers on a 5-point Likert scale (1 = very good, 2 = good, 3 = satisfactory, 4 = poor, and 5 = very poor). For some analyses the items were dichotomized from very good/good and satisfactory to (very) poor.

### 2.4. Statistical Analyses

Descriptive analyses were applied to describe the core characteristics of the included children and adolescents. The assumptions of normal distribution were checked through the Shapiro–Wilk test. As none of the examined variables were normally distributed (all *p* < 0.05), nonparametric tests were applied. Spearman–Rho correlations identified the relationships between the five SNAKE scales and the global rating, as well as between children’s age and the sleep ratings. A correlation coefficient between 0.1 and 0.3 shows a weak association, whereas a coefficient between 0.3 and 0.5 reflects a medium association and a coefficient above 0.5 is indicative of a strong association. A Mann–Whitney U test was conducted and adjusted by Bonferroni correction to see if there were significant differences between the two dichotomized groups (global rating; very good/good–satisfactory to (very) poor) for the five SNAKE scales. The significance level was set at *p* = 0.05 (two-tailed). All analyses were conducted using SPSS (version 25, IBM, Chicago, IL, USA).

## 3. Results

### 3.1. Sample Characteristics

Of the *N* = 226 children and adolescents, *n* = 14 had to be excluded (*n* = 12 core parameters incomplete/more than 50% missing; *n* = 2 SNAKE incomplete/more than 50% missing). The final sample consisted of *n* = 212 children and adolescents (*n* = 99, 46.7% female; *n* = 113, 53.3% male) aged between 1 and 25 years (*M* = 10.4; SD = 5.5). Cerebral palsy (*n* = 54, 26.3%), global developmental retardation (*n* = 35, 17.1%), different rare syndromes (*n* = 28, 13.7%) and neurodegenerative diseases/metabolic disorders (*n* = 21, 10.2%) were the most common diagnoses in this sample.

The SNAKE was completed by the mother for *n* = 187 (88.2%) children and by the father for *n* = 14 (6.6%) children (mother and father together: *n* = 5, others: *n* = 6).

Referring to the global sleep rating, *n* = 42 (19.8%) children were rated as having very good, *n* = 67 (31.6%) good, *n* = 70 (33%) satisfactory, *n* = 26 (12.3%) poor, and *n* = 7 (3.3%) very poor sleep during the past four weeks. Children were nearly equally allocated to the two dichotomized groups that reflected the child’s general sleep quality (very good/good: *n* = 109, 51.4%; satisfactory to (very) poor: *n* = 103, 48.6%).

### 3.2. Relationship between the Global Rating of a Child’s Sleep and the SNAKE Scales

Table 1 demonstrates the Spearman–Rho correlations between the SNAKE scales and the global sleep rating (all *p* < 0.01). It is clear that each of the five scales correlates positively and significantly with the global rating. The association with the global rating is strong for disturbances in terms of going to sleep and disturbances with respect to remaining asleep, medium for arousal and breathing disorders as well as daytime behavior disorders, and weak for daytime sleepiness.

**Figure 1:**
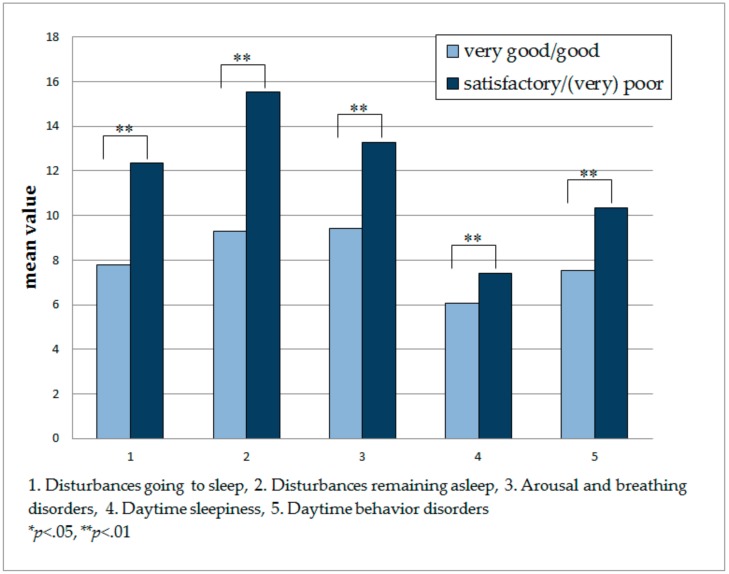
Differences between global sleep quality in the two subgroups for all five scales of the Sleep Questionnaire for Children with Severe Psychomotor Impairment (SNAKE).

Mann–Whitney U testing revealed that children who were classified as having a satisfactory to (very) poor sleep during the past four weeks scored significantly higher in all five SNAKE scales as compared to children who were classified as having a very good/good sleep during the abovementioned time frame (Figure 1). This result was also confirmed after Bonferroni correction.

### 3.3. Relationship between Age and Sleep Ratings

Because of the broad age range of children and adolescents that were included in analyses, we further tested if there were any relationships between children’s age and the reported sleep problems. “Disturbances going to sleep” (*r* = −0.14, *p* < 0.05), “Daytime sleepiness” (*r* = −0.18, *p* < 0.01) and “Daytime behavior disorders” (*r* = −0.23, *p* < 0.01) correlated negatively and significantly with children’s age, i.e., the younger the child, the stronger the sleep disturbance. The remaining two SNAKE scales (“Disturbances remaining asleep” and “Arousal and breathing disorders”) and the global rating of a child’s sleep did not correlate with children’s age (all *p* > 0.05).

## 4. Discussion

Results show that the two examined sources of information, the global rating of a child’s sleep, which directly measures the parents’ assessment of their child’s sleep quality, and the five SNAKE scales, which indirectly measure the child’s sleep behavior, are consistent. The highest concordance with the global rating was achieved for disturbances going to sleep and disturbances remaining asleep.

Analyses reveal positive correlations between all five scales and the global rating. Even though this result must not be interpreted as evidence of causality, it is an important clue that both sources of information tend towards the same direction. This assumption is also supported by the fact that in comparison with children who slept very well or well during the past four weeks, children who were identified as having slept only satisfactorily, poorly or very poorly during that period concurrently obtained significantly higher values for each of the five SNAKE scales and therefore suffered from more sleep problems than children in the other group. This result emphasizes, once more, that the scales seem to be appropriate for mapping sleep(-related) problems in the vulnerable group of children with LLCs and SPMI [21]. Interestingly, the differences between the mean values of the two groups (very good/good; satisfactory to (very) poor) differed between the five scales: They were the largest for scale 2 (disturbances remaining asleep) and the smallest for scale 4 (daytime sleepiness). Thus, some specific aspects of sleep problems in children with LLCs and SPMI seem to distinguish between children with good and poor sleep more strongly than others. Nonetheless, as our questioning and the analyses we conducted do not allow any conclusions to be drawn about the causality of the relationship, we cannot clearly interpret this finding and instead defer to future studies. Three of the five SNAKE scales, but not the global rating of a child’s sleep, correlate negatively with children’s age. That result indicates that severely disabled children’s specific sleep problems decline with children’s increasing age; this has also been shown in prior studies [13]. Another explanation could be that parents monitor their child’s sleep more intensively when he or she is younger and therefore can make a more precise report on specific sleep problems than parents of older children. Nevertheless, we did not find a relationship between a child’s sleep problems in general and the child’s age. There are also studies that did not find or do not assume such a decline of sleep problems with increasing age [10,19]. It must be considered that the actual age of children and adolescents with LLCs and SPMI commonly differs from their developmental age, which might be a reason for our inconclusive result. Therefore, investigations are needed to better understand the impact of children’s age or developmental age on sleep problems.

In nearly 90% of the cases included in this study, mothers completed the questionnaire. As the mother is often the primary caretaker of their ill child [18,19,33], it can be expected that in general their statements are reliable because of the expertise they have regarding their child’s general condition and their child’s sleep. Nevertheless, it must be noted that our data are highly subjective and should therefore be underpinned with objective measures such as polysomnography or actigraphy in future research efforts.

In the current sample, children and adolescents of different ages and with different diagnoses are represented. It can therefore be concluded that our results are valid for a broad range of severely disabled children. Nevertheless, we did not consider special characteristics that go along with the children’s diagnoses (e.g., need for repositioning, use of ventilation); this additional information would be helpful to refine our findings. Furthermore, we did not compare our results for different groups of diagnoses. Within the framework of this study, this must not be seen as a deficiency because our aim was to make a general statement on the SNAKE’s methodological quality. Notwithstanding, the comparison of sleep characteristics of children with different life-limiting diagnoses would be an interesting approach for future studies in general. The global sleep rating of the children and adolescents included in this study was mainly in the “very good”, “good”, or “satisfactory” range. This result is different from studies that describe a high range of sleep problems in severely disabled children [4]. A reason for that could be that the LLC cohort includes a very heterogeneous group of children and adolescents with various illnesses, comorbidities, and personal characteristics [34]. Therefore, a direct comparison between prevalence rates of different studies on that heterogeneous population is only feasible to a limited extent [35].

A clear limitation of the SNAKE is its lack of cutoff values [21], which makes it impossible to state whether the values the included children reached for the five SNAKE scales are clinically meaningful or not. The implementation of cutoff values is urgently needed to strengthen the usefulness of the SNAKE in clinical contexts.

In summary, the five scales of the SNAKE that indirectly assess different aspects of sleep(-related) problems seem to correspond with the parent’s direct judgment on their child’s sleep quality. Therefore, our results are additional proof that the SNAKE is a valid questionnaire for assessing sleep problems in severely disabled children. Furthermore, it underscores that in contrast with other pediatric sleep questionnaires [22,23,24], the SNAKE meets the challenges of children with LLCs and SPMI. Future research should primarily address the development of cutoff values for the SNAKE and the inclusion of objective measures. These efforts would advance knowledge regarding sleep problems in this vulnerable population.

## Figures and Tables

**Table 1:** Correlations between five Sleep Questionnaire for Children with Severe Psychomotor Impairment (SNAKE) scales and the global rating of a child’s sleep.

Measure	1	2	3	4	5
1. Disturbances going to sleep	-	-	-	-	-
2. Disturbances remaining asleep	0.57 **	-	-	-	-
3. Arousal and breathing disorders	0.34 **	0.45 **	-	-	-
4. Daytime sleepiness	0.10	0.36 **	0.37 **	-	-
5. Daytime behavior disorders	0.45 **	0.50 **	0.30 **	0.14 *	-
6. Global sleep rating	0.61 **	0.73 **	0.41 **	0.23 **	0.44 **

* *p* < 0.05, ** *p* < 0.01.
